# Mechanisms of cordycepin in the treatment of pulmonary arterial hypertension in rats based on metabonomics and transcriptomics

**DOI:** 10.1038/s41598-024-62163-3

**Published:** 2024-05-30

**Authors:** Jiangpeng Lin, Riken Chen, Huizhao Liao, Yuzhuo Zhang, Zhenzhen Zheng, Cheng Hong

**Affiliations:** 1 Department of Respiratory and Critical Care Medicine, The Second Affiliated Hospital of Guangdong Medical University, Zhanjiang, 524003 Guangdong China; 2 Guangzhou Medical University, State Key Laboratory of Respiratory Disease, National Clinical Research Center for Respiratory Disease, Guangzhou Institute of Respiratory Health, The First Affiliated Hospital of Guangzhou Medical University, Guangzhou, 510120 Guangdong China; 3 National Cancer Center/National Clinical Research Center for Cancer/Cancer Hospital, Chinese Academy of Medical Sciences and Peking Union Medical College, Beijing, 100021 China; 4 Nanshan School, Guangzhou Medical University, Guangzhou, 511436 China

**Keywords:** Cordycepin, Pulmonary arterial hypertension, Metabolomics, Transcriptomics, P53, P21, Pharmacology, Computational biology and bioinformatics, Drug discovery, Molecular biology, Medical research

## Abstract

Pulmonary arterial hypertension (PAH) is a fatal disease featured by high morbidity and mortality. Although Cordycepin is known for its anti-inflammatory, antioxidant and immune-enhancing effects, its role in PAH treatment and the underlying mechanisms remain unclear. The therapeutic effects of Cordycepin on rats with PAH were investigated using a monocrotaline (MCT)-induced rat model. The metabolic effects of Cordycepin were assessed based on the plasma metabolome. The potential mechanisms of Cordycepin in PAH treatment were investigated through transcriptome sequencing and validated in pulmonary artery smooth muscle cells (PASMC). Evaluations included hematoxylin and eosin staining for pulmonary vascular remodeling, CCK-8 assay, EDU, and TUNEL kits for cell viability, proliferation, and apoptosis, respectively, and western blot for protein expression. Cordycepin significantly reduced right ventricular systolic pressure (RVSP) and right ventricular hypertrophy index (RVHI) in PAH rats, and mitigated pulmonary vascular remodeling. Plasma metabolomics showed that Cordycepin could reverse the metabolic disorders in the lungs of MCT-induced PAH rats, particularly impacting linoleic acid and alpha-linolenic acid metabolism pathways. Transcriptomics revealed that the P53 pathway might be the primary pathway involved, and western blot results showed that Cordycepin significantly increased P53 and P21 protein levels in lung tissues. Integrated analysis of transcriptomics and metabolomics suggested that these pathways were mainly enriched in linoleic acid metabolism and alpha-linolenic acid metabolism pathway. In vitro experiments demonstrated that Cordycepin significantly inhibited the PDGFBB (PD)-induced abnormal proliferation and migration of PASMC and promoted PD-induced apoptosis. Meanwhile, Cordycepin enhanced the expression levels of P53 and P21 proteins in PD-insulted PASMC. However, inhibitors of P53 and P21 eliminated these effects of Cordycepin. Cordycepin may activate the P53–P21 pathway to inhibit abnormal proliferation and migration of PASMC and promote apoptosis, offering a potential approach for PAH treatment.

## Introduction

Pulmonary arterial hypertension (PAH) is a progressive disease that increases pulmonary artery pressure and pulmonary vascular resistance, leading to right heart failure and death^1^. Recent studies indicate that PAH affects approximately 1% of the global population and up to 10% in those over 65 years old^2^. The pathophysiology of PAH involves abnormal pulmonary artery contraction, pulmonary inflammation, vascular remodeling, thrombosis, abnormal proliferation and migration of pulmonary artery smooth muscle cells (PASMC). Experts believe that abnormal proliferation and migration of PASMC significantly impact PAH, leading to pulmonary vascular remodeling^3^. The primary treatment for PAH involves drug treatment. While these drugs can decelerate the progression of the disease or alleviate its symptoms, they are associated with serious side effects, high costs, and a substantial rate of recurrence^4^. Consequently, it is critical to investigate the underlying molecular mechanisms of PAH and identify novel, effective treatments for pulmonary arterial hypertension.

Chinese medicine is known globally for its ability to boost immunity and regulate systemic health, while also being relatively safe. Cordycepin (Cor), also known as 3ʹ-deoxyadenosine, is the active ingredient unique to Chrysoprase^5^. The nutritional and chemical compositions, pharmacological properties, and clinical effects of Chrysalis are essentially identical to those of Cordyceps, which was historically used in ancient China to protect the lungs and benefit the kidneys, regulate bodily functions, treat back and knee pain, and alleviate bleeding and phlegm. Since the therapeutic effects of Chrysalis are not statistically different from those of wild Cordyceps, it is not prohibitively expensive. Cordycepin has exhibited anti-tumor, anti-leukemia, antibacterial, anti-inflammatory, and anti-aging properties in various studies^6,7^. Yang Chunsheng and colleagues reported that Cordycepin significantly reduced the expression of MMP-2, MMP-9, and MMP-13 in platelet-derived growth factor-induced smooth muscle cells in rats, thereby preventing atherosclerosis^8^. High concentrations of Cordycepin (40 g/ml) inhibited the inflammatory response induced by lipopolysaccharide^9^. Zheng et al. found that Cordycepin increased the expression of BAX and Caspase 3 while decreasing Bcl-2 expression at both mRNA and protein levels^10^. Research suggests that Cordycepin could potentially treat pulmonary arterial hypertension by reducing lung inflammation, inhibiting abnormal value addition and migration of PASMC, and preventing remodeling of small pulmonary vessels.

In the current study, pulmonary arterial hypertension in rats was induced using Monocrotaline (MCT)^11^. We focused on plasma metabolomics to assess the efficacy of Cordycepin in treating pulmonary arterial hypertension. Additionally, transcriptome sequencing was employed to investigate the potential mechanisms of Cordycepin’s therapeutic effects and to validate these findings in pulmonary artery smooth muscle cells.

## Material and methods

### Reagents

Cordycepin with a purity greater than 98% was provided by Chengdu Must Bio-Technology Co., Ltd. (CAS 73-03-0). Monocrotaline (MCT) (CAS:315-22-0) was obtained from MedChemExpress (MCE). Inhibitors of P53 and P21 were also provided by MCE. The bicinchoninic acid (BCA) protein assay kit (No.: KGP902), EDU Kit (No.: KGA331-100) and TUNEL Kit (No.: KGA7071) were purchased from KeyGen Biotech (Jiangsu). Antibodies including PCNA, BAX, Bcl-2, Caspase 3, P53 P21, α-SMA and GAPDH were provided by Abcam.

### Animals and experimental design

**Figure 1:**
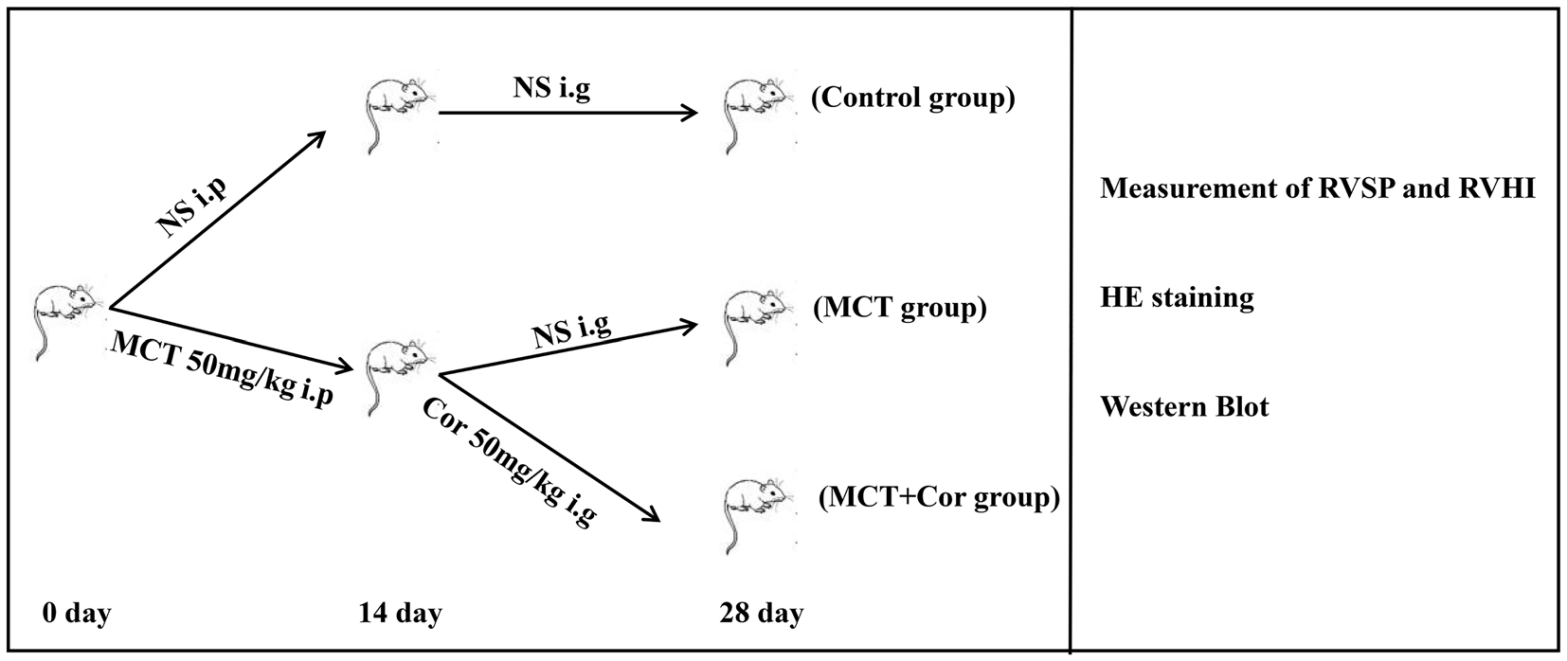
Flow chart of animal experiments. i.p., intraperitoneal injection; i.e., intragastric administration.

Male Sprague-Dawley (SD) rats (180–220 g, 6–8 weeks old, healthy and energetic) were used for this study under the support of Guangdong Medical Laboratory Animal Center. The experimental protocol for animal experimentation was approved by the First Hospital of Guangzhou Medical University (Ethical Review No.: 2022014). The study adhered strictly to the guidelines in the National Institutes of Health Guide for the Care and Use of Laboratory Animals and compliance with the ARRIVE guidelines was confirmed. All rats were maintained under a 12 h light/12 h dark cycle at 22 ± 3 °C with free access to water and food. The animals were randomly divided into three groups (n = 5 per group): (1) Control group; (2) MCT group; and (3) MCT + Cor group. PAH was induced in the MCT and MCT + Cor groups through intraperitoneal injection of 50 mg/kg MCT solution. The Control group received a comparable volume of normal saline intraperitoneally. Fourteen days post MCT injection, once severe PAH was established, the MCT + Cor group were given Cordycepin 50 mg/kg daily via intragastric administration following the methodology of Su et al.^12^. Both the Control and MCT groups were given the quivalent normal saline. The flow chart illustrating the animal experimental design is shown in Fig. 1.

### Measurement of the right ventricular systolic pressure (RVSP) and right ventricular hypertrophy index (RVHI)

After 28 days of administration, rats were anesthetized with an animal anesthesia machine. To measure RVSP, a catheter was inserted into the right atrium through the right external jugular vein and advanced 1 cm forward. The catheter was then rotated 90° counterclockwise to position the tip within the right ventricle. For assessing right ventricular hypertrophy, the heart was anatomically divided into three chambers: the right ventricle (RV), the left ventricle (LV), and the septum (S). The RVHI was calculated using the formula: RVHI = weight of RV/(weight of LV + S).

### Arterial histological assessment

Before dehydration, rat lung tissue samples were fixed in formalin for a minimum of 48 h. Subsequently, the tissues were embedded in paraffin, sectioned into 5 μm slices, and stained with HE. These sections were then scanned using the ImageScope scanning system. In this single-blind study, twenty-three pulmonary arteries, with diameters ranging from 50 to 150 μm, were randomly selected from each rat for evaluation. A pathologist observed and analyzed these sections. Two key indices of pulmonary remodeling were calculated for each sample: (1) pulmonary wall thickening = (outer diameter − inner diameter)/outer diameter; (2) pulmonary wall area = (transection area of walls − lumen area)/transection area of walls.

### Ultra performance liquid chromatography/tandem mass spectrometry (UPLC-MS/MS) assay

Untargeted metabolomics was performed using plasma samples from five rats in the Control group and four rats in each of the MCT and MCT + Cor groups. For each sample, 100 μL plasma sample was mixed with 400 μL extraction solution (acetonitrile:methanol = 1:1) containing 0.02 mg/mL internal standard (l-2-chlorophenylalanine), and subjected to ultrasonic extraction at low temperature (5 °C, 40 kHz) for 30 min. The samples were then centrifuged at 4 °C and 13,000*g* for 15 min; the supernatant was removed, and the residue was redissolved in 100 µL of a solution (acetonitrile:water = 1:1). This mixture underwent a second low-temperature ultrasonic extraction for 5 min (5 °C, 40 kHz), followed by centrifugation at 4 °C, 13,000*g* for 10 min to remove the supernatant for subsequent analysis.

The prepared samples were analyzed using an UPLC-MS/MS system, specifically the ultra-high performance liquid chromatography tandem Fourier transform mass spectrometry (UHPLC-QEXactiveHF-X). The chromatographic conditions employed a 3 μL sample injection through an HSST3 column. The mobile phases used were: Mobile phase A, consisting of 95% water + 5% acetonitrile; and Mobile phase B, consisting of 47.5% acetonitrile + 47.5% isopropyl alcohol + 5% water. The mass spectrum conditions included both positive and negative ion scanning modes with a mass scanning range of 70–1050 m/z. The sheath gas flow rate was set at 50 psi, the auxiliary gas flow rate at 13 psi, and the auxiliary gas heating temperature at 425 °C. The positive mode ion spray voltage was set to 3500 V and the negative mode to − 3500 V. The ion transport tube temperature was maintained at 325 °C, and the normalized collision energy was cycled at 20–40–60 V. The primary mass spectral resolution was 60,000, the secondary mass spectral resolution was 7500, and data were collected in DDA mode.

After the computer was configured, the original LC–MS data was imported into the metabolomics processing software ProgenesisQI (Waters Corporation, Milford, USA) for baseline filtering, peak identification, integration, retention time correction, and peak alignment. This processing resulted in a data matrix consisting of retention time, mass-to-charge ratio, and peak intensity values. The data were then cross-referenced with the HMDB mass spectrometry and metabolic information public database (http://www.hmdb.ca/) and Metlin match (https://metlin.scripps.edu) for metabolite identification.

Subsequent analysis involved principal component analysis (PCA) and orthogonal least partial square discriminant analysis (OPLS-DA) on the obtained data matrix, where the stability of the model was evaluated through seven cycles of interactive verification. Metabolites showing significant differences were identified based on the Variable Importance in Projection (VIP) values obtained from the OPLS-DA model and the corresponding P-values; metabolites with VIP values greater than 1 and P-values less than 0.05 were considered to significantly differentiate between groups. The metabolic pathways involving these differentially expressed metabolites were annotated using the Kyoto Encyclopedia of Genes and Genomes (KEGG) database (https://www.kegg.jp/kegg/pathway.html).

### Transcriptome sequencing

Transcriptome sequencing was conducted on four randomly selected rats from each of the Control, MCT, and MCT + Cor groups. The sequencing was performed by Shanghai Mayobio Biopharmaceutical Technology Co., Ltd. Total RNA was extracted from the left lung tissues using a Trizol kit (Invitrogen, Carlsbad, CA, United States). The quality of the extracted RNA was assessed with an Agilent 2100 Bioanalyzer (Agilent Technologies, Palo Alto, CA, United States) and further verified through RNase-free agarose gel electrophoresis. After total RNA extraction, the RNA was enriched using Oligo (dT) beads and reverse transcribed into cDNA using random primers. The second strand of cDNA was then synthesized, purified, end-repaired, and ligated to Illumina sequencing adapters. The size of the ligated products was selected through agarose electrophoresis. PCR amplification was subsequently performed, and the samples were sequenced using an Illumina HiSeq2500 system provided by Gene Denovo Biotechnology Co., Ltd. (Guangzhou, China). The analysis strictly adhered to established protocols to identify differentially expressed genes, with significant changes defined by a log (FC) > 1 and P < 0.05. Gene Ontology (GO) and KEGG pathway enrichment analyses were performed using the DAVID (https://david.ncifcrf.gov/).

### Integrated analysis of transcriptomics and metabolomics

To perform a comprehensive integrated analysis of the metabolomics and transcriptomics data, we employed the Joint-Pathway Analysis tool available in MetaboAnalysis 5.0 (https://new.metaboanalyst.ca/MetaboAnalyst). Differentially expressed genes and metabolites were entered into the platform, specifying Rattus norvegicus as the species. The analysis was conducted with the threshold for statistical significance set at P < 0.05.

### Cell culture

Six-week-old male SD rats were anesthetized by intraperitoneal injection with sodium pentobarbital (100 mg/kg) and then euthanized with CO_2_. The middle layer of the pulmonary artery was quickly and carefully removed under the microscope. PASMC were isolated using tissue-adhesive collagenase digestion. Primary PASMC were cultured in DMEM/F12 medium containing 0.5% streptomycin, 0.5% penicillin and 10% fetal bovine serum, and maintained in a 37°C incubator with 5% CO_2_. The medium was changed every two days to maintain cell viability. The cells were passaged upon reaching 90% confluency. PASMC from the 3rd to 5th generation were used in this study. Prior to any experimental intervention, these cells were serum-starved for 24 h to synchronize their growth phase. PASMC identification was confirmed by immunofluorescence, using an anti-α-smooth muscle actin (α-SMA) antibody, and the cells' morphology was examined under a fluorescence microscope (details provided in Supplementary Material 1).

### Cell viability assay screening for optimal experimental conditions with CCK-8

To determine the optimal concentration of the drug for the experiments, a range of drug concentration gradients were tested: 0, 5, 10, 20, 50, 100, and 200 μM. Each group, including these various concentrations, received 20 ng/ml of PDGFBB (PD) and was incubated for 24 h. For comparison and control purposes, a control group containing only the cell culture medium and a blank group devoid of both cells and drug medium were established. The absorbance at 450 nm was measured using a microplate reader.

### Cell migration analysis

To assess cell migration, PASMC cultures reaching 90% confluence were serum-starved with 0% fetal bovine serum (FBS) for 24 h to synchronize their growth phase. The following day, a wound healing assay was conducted. A straight line was drawn with a ruler across the cell monolayer in a six-well plate using a 200 µl pipette tip to create a "scratch." The cells were then washed three times with PBS. Cells from the control group, the PD group (20 ng/ml PD), and the PD + Cor group (20 ng/ml PD + 50 µM Cor) were cultured in their respective media. The initial cell density along the scratch was recorded at 0 h. After 24 h, the cells were photographed again to assess migration into the scratched area. The rate of cell migration was quantified by measuring the change in the scratch zone area from 0 to 24 h using ImageJ software. The cell migration rate was calculated using the formula: Migration rate = (scratch zone at 0 h − scratch zone at 48 h)/scratch zone at 0 h.

### Measurement of cell proliferation

EDU kits was used to determine cell proliferation in this study. The experiment included several groups: Control group, PD group, PD + Cor group, PD + Cor + P53 inhibitor group, and PD + Cor + P21 inhibitor group. The cells were incubated with EDU, then fixed by osmosis, and subsequently stimulated. The incorporation of EDU into the cells was observed using a fluorescence microscope. Cell images were captured and analyzed using Image J software. The proportion of actively dividing cells (positive cells to total cells ratio) was calculated.

### Apoptosis assay

TUNEL kit is a common method to detect apoptosis. Cells were cultured to 90% density and treated with 0% FBS starvation for 24 h. The experimental groups were designed as follows: Control group, PD group, PD + Cor group, PD + Cor + P53 inhibitor group, and PD + Cor + P21 inhibitor group. The TUNEL kit was used to detect the breakage of nuclear DNA during apoptosis. The TUNEL positive cell rate (positive cells/all cells) was photographed and analyzed using ImageJ software.

### Western blot

Lysis was performed by adding 50 mg of large lung tissue or PASMC to 500 all of pre-cooled RIPA lysate (100:1:1 RIPA, PMSF and phosphatase inhibitor lysate before use). The total protein content in the lysate was determined by the BCA method. Subsequently, 30 μg of total protein lysate subjected to 10% SDS-PAGE and transferred onto nitrocellulose membranes, which were then blocked with 5% skim milk powder and incubated overnight at 4 °C. Primary antibodies were PCNA (1:2000), BAX (1:2000), Bcl-2 (1:1000), Caspase3 (1:2000), P53 (1:2000), and P21 (1:2000). Following primary antibody incubation, the membranes were exposed to IgG-HRP secondary antibody (1:5000) for 2 h at room temperature. The protein bands were visualized using an ECL luminescent solution, and grayscale values were measured with ImageJ software to quantify protein expression levels. All protein bands are detailed in Supplementary Material 2. Note that the blotting membranes were cut prior to hybridization with antibodies to conserve time and reagents. According to the target protein sizes, only the relevant strips were cut for hybridization with the corresponding antibodies. All procedures were rigorously followed and repeated five times to ensure reliability and reproducibility of the results.

### Molecular docking

To further investigate the relationship between Cordycepin and PAH, molecular docking studies were conducted. The 3-D structures of the target proteins (P53 and P21) and Cordycepin were retrieved from the RCSB Protein Data Bank (https://www.rcsb.org) and the PubChem database (https://pubchem.ncbi.nlm.nih.gov), respectively. Using PyMOL, the proteins were prepared by dehydration, and their charges and hydrogen atoms were added using AutoDock software. Molecular docking was performed with AutoDock Vina to identify the optimal binding conformation and calculate the hydrogen bond lengths between Cordycepin and the proteins, providing insights into the potential molecular interactions.

### Statistical analysis

All data were expressed as mean ± standard deviation (SD), and statistical analysis was performed using SPSS 24.0 statistical software. All experiments were repeated at least three times. The data were analyzed by one-way ANOVA (one-way ANOVA) to determine the differences between two groups. For all statistical tests, p < 0.05 was considered statistically significant.

### Institutional Review Board Statement

The study was approved by the Ethics Committee of the First Affiliated Hospital of Guangzhou Medical University (No. 2022014). The study was conducted in strict accordance with the guidelines in the National Institutes of Health Guide for the Care and Use of Laboratory Animals. And we confirmed that the authors strictly complied with the ARRIVE guidelines.

## Results

### Cordycepin improves hemodynamics and pulmonary vascular remodeling in rats with pulmonary arterial hypertension

**Figure 2:**
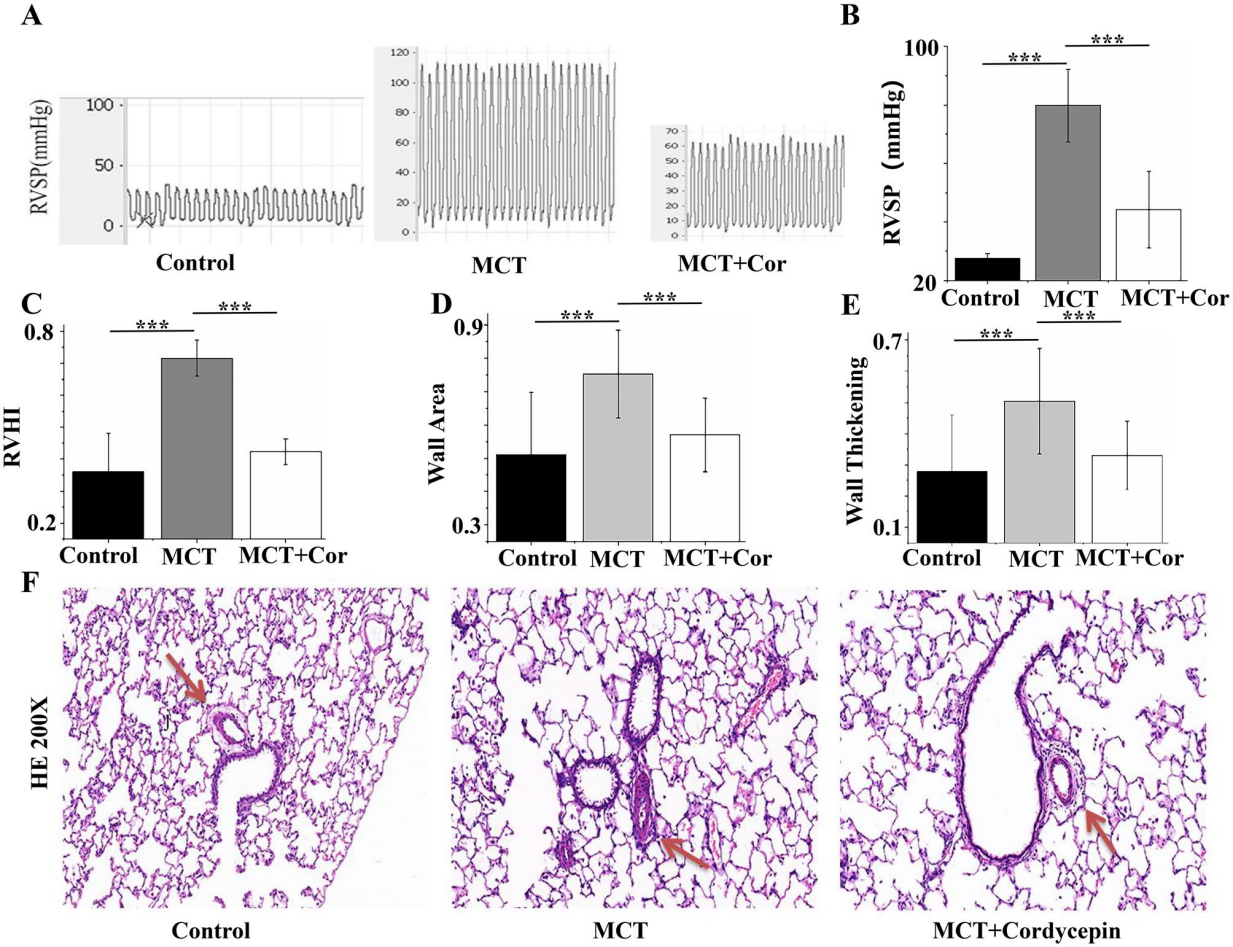
Cordycepin effectively improved MCT-induced pulmonary arterial hypertension and pulmonary vascular remodeling in rats. (**A**) Representative images of RVSP waveforms; (**B**) Statistical graph of RVSP (n = 5); (**C**) Statistical results of RVHI (n = 5); (**D**) Statistical results of pulmonary artery Wall Area ratio. (**E**) Statistical results of pulmonary artery wall thickening. (**F**) Pulmonary artery HE staining in Control group, MCT group and MCT + Cor group (200 × magnification, n = 23). ***p < 0.001.

MCT-induced RVSP and RVHI were significantly elevated in PAH rats compared to control rats. Cordycepin treatment (50 mg/kg) for two weeks significantly reduced both RVSP and RVHI in these PAH rats (Fig. 2A–C). Histological analyses through HE staining showed extensive pulmonary vascular remodeling, with increased lung wall thickening and lung wall area in PAH rats. Cordycepin treatment effectively inhibited these changes, significantly reducing pulmonary vascular remodeling, lung wall thickening, and lung wall area (Fig. 2D–F). Taken together, these findings suggest that Cordycepin can effectively alleviate MCT-induced pulmonary arterial hypertension and pulmonary vascular remodeling in rats.

### Cordycepin reverses MCT-induced metabolic disorders in the lungs of rats with pulmonary arterial hypertension

**Figure 3:**
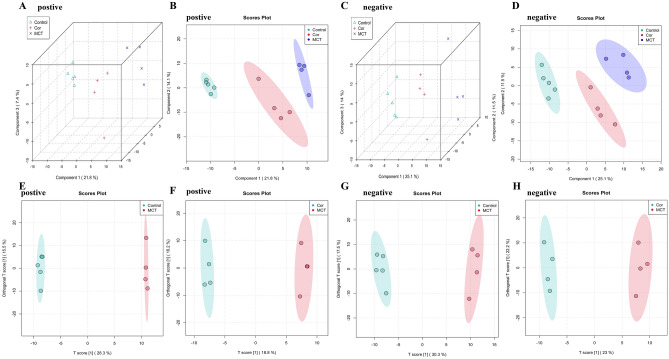
Different groups' sample distributions in PCA, PLS-DA, and OPLS-DA analysis modes. (**A**) Positive ion mode PCA 3D distribution; (**B**) Positive ion mode PLS-DA score plot; (**C**) Negative ion mode PCA 3D distribution; (**D**) Positive ion mode PLS-DA score plot; (**E**) Positive ion mode OPLS-DA mode sample distribution for Control and MCT groups; (**F**) Negative ion mode Control and MCT groups OPLS-DA mode sample distribution map; (**G**) OPLS-DA mode sample distribution map of MCT group and MCT + Cor group in positive ion mode; (**H**) OPLS-DA mode sample distribution map of MCT group and MCT + Cor group in negative ion mode.

In order to assess the impact of Cordycepin on metabolic disruptions in PAH rats, plasma samples from the Control, MCT, and MCT + Cor groups were analyzed under positive and negative ion patterns. The PCA score plots (Fig. 3A,C) display the distribution of the three groups within a 3D space, with the variables demonstrating biological relevance within the 95% confidence interval and samples from each group closely clustered. Further analysis using Partial Least Squares Discriminant Analysis (PLS-DA) indicated significant clustering for both ion patterns among the groups, highlighting pronounced inter-group metabolic differences over intra-group variations (Fig. 3B,D). Moreover, the model group exhibited clear metabolic distinctions from the control group, suggesting that MCT induces significant metabolic alterations. The MCT + Cor group showed metabolic profiles trending towards those of the control group, suggesting that Cordycepin effectively blocks the metabolic disturbances induced by MCT. To elucidate the mechanisms by which Cordycepin counteracts PAH-induced metabolic disruptions, Orthogonal PLS-DA (OPLS-DA) was employed to identify specific ion expression differences among the Control, MCT, and MCT + Cor groups. This analysis further differentiated the groups in both positive and negative modes (Fig. 3E–H). Comparisons between the control and MCT groups exhibited high model fidelity and predictive reliability, with R2Y = 0.999 and Q2 = 0.907 in the positive ion mode, and R2Y = 0.994 and Q2 = 0.883 in the negative ion mode. Comparisons between MCT and MCT + Cor groups in the positive ion mode resulted in R2Y = 0.996 and Q2 = 0.6, and in the negative ion mode, R2Y = 0.994 and Q2 = 0.696, indicating optimal model performance. The results indicate that the model has solid fitting and predictive capabilities without overfitting, making it suitable for screening differential markers.

### Screening of potential metabolic markers and analysis of differential metabolic pathways

**Figure 4:**
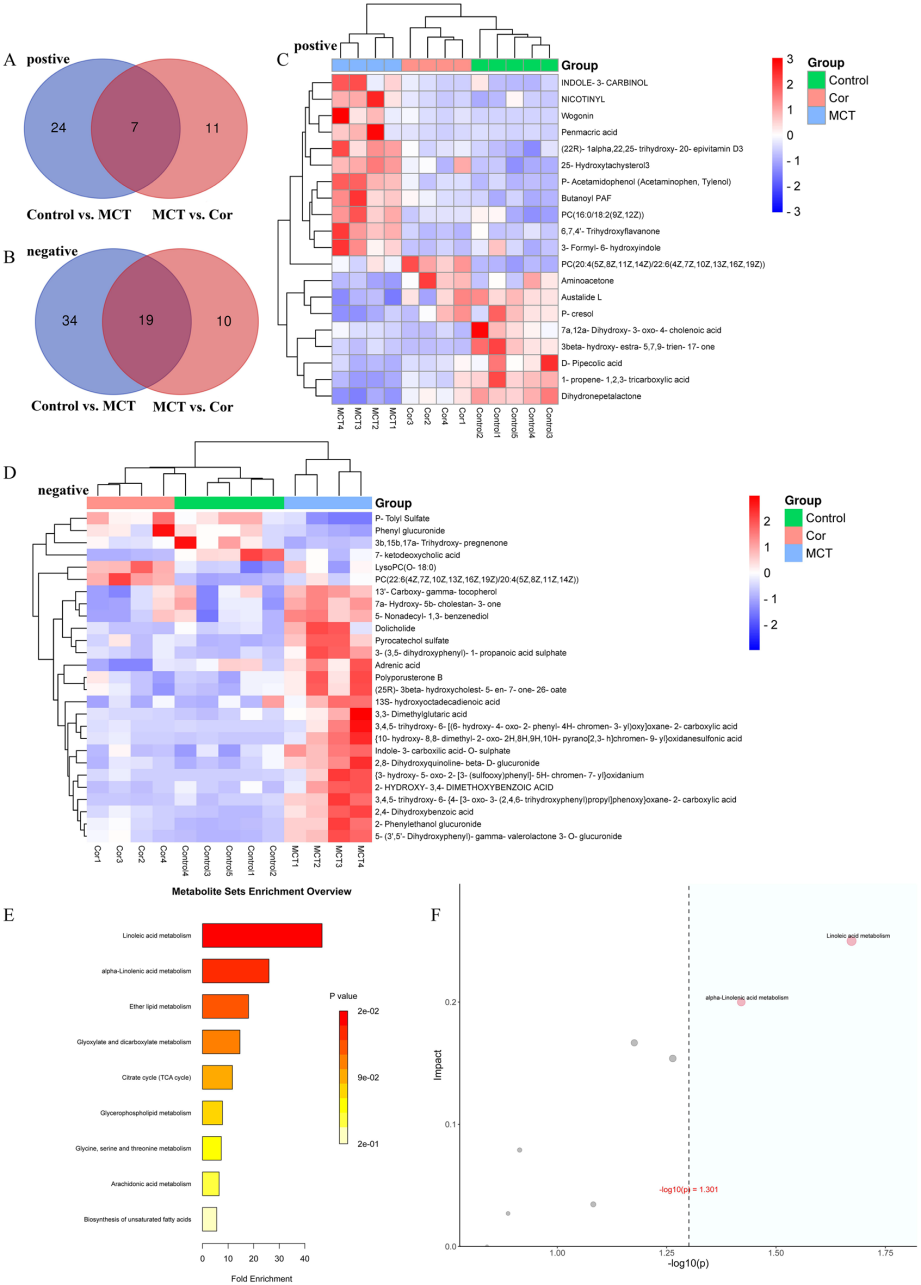
Analysis of differential metabolic pathways and screening for potential metabolic markers. (**A**) Positive ion mode Venn diagram of metabolites in different groups; (**B**) Negative ion mode Venn diagram of metabolites in different groups; (**C**) Positive ion mode heat map of differential metabolite correlation; (**D**) Heat map of differential metabolite correlation in negative ion mode; (**E**) Enrichment analysis of MCT + Cor group and MCT group; (**F**) Topological analysis of MCT + Cor group and MCT group. F-plot horizontal coordinates are ORA analysis p-values, blue areas are significant (p < 0.05). Vertical coordinates are the most significant results of topological analysis.

In the analysis of metabolic alterations, we identified 105 differentially expressed metabolites from the Control, MCT, and MCT + Cor groups using both positive and negative ion modes (Fig. 4). Specifically, 31 metabolites showed differential expression in the MCT group compared to the Control group in positive ion mode, as depicted in the Venn diagram, while 53 metabolites displayed significant changes in negative ion mode. Following the administration of Cordycepin to PAH rats, plasma levels of 7 metabolites normalized in positive ion mode, and 19 metabolites returned to normal in negative ion mode (Fig. 4A,B).

To further investigate the impact of Cordycepin on the metabolic profiles of rats with pulmonary arterial hypertension, the OPLS-DA method was used to analyze differences between groups. Metabolites were selected based on their variable importance projection (VIP) values and p-values, with those showing VIP > 1 and p < 0.05 considered significant contributors to clustering and differentiation. This approach identified 47 distinct metabolites meeting the screening criteria (Table 1).

**Table 1:** Differential metabolites in plasma samples in positive and negative mode.

Mode	Metabolite	Formula	m/z	Retention time	Control vs. MCT	MCT vs. Cor
pos	(22R)-1alpha,22,25-trihydroxy-20-epivitamin D3	C27H44O4	433.3299527	6.5901	Down	Down
pos	1-Propene-1,2,3-tricarboxylic acid	C6H6O6	139.0023662	1.37665	Up	Up
pos	25-Hydroxytachysterol3	C27H44O2	401.3406268	9.418716667	Down	Down
pos	3beta-hydroxy-estra-5,7,9-trien-17-one	C18H22O2	271.1684847	5.7416	Up	Down
pos	3-Formyl-6-hydroxyindole	C9H7NO2	162.0547273	2.31735	Down	Down
pos	6,7,4ʹ-Trihydroxyflavanone	C15H12O5	273.0752441	3.4408	Down	Down
pos	7a,12a-Dihydroxy-3-oxo-4-cholenoic acid	C24H36O5	387.2521459	5.981583333	Up	Down
pos	Aminoacetone	C3H7NO	137.0706247	0.659083333	Up	Up
pos	Austalide L	C25H32O6	446.2527289	6.354333333	Up	Up
pos	Butanoyl PAF	C28H58NO7P	552.4013789	9.312316667	Down	Down
pos	Dihydronepetalactone	C10H16O2	169.1219662	9.611516667	Up	Up
pos	d-Pipecolic acid	C6H11NO2	130.0860958	0.541983333	Up	Up
pos	INDOLE-3-CARBINOL	C9H9NO	148.0755257	3.598883333	Down	Down
pos	NICOTINYL	C6H7NO	110.0601666	2.161416667	Down	Down
pos	P-Acetamidophenol (Acetaminophen, Tylenol)	C8H9NO2	152.0704899	2.1838	Down	Down
pos	PC (16:0/18:2(9Z,12Z))	C42H80NO8P	758.567784	11.87875	Down	Down
pos	PC (20:4(5Z,8Z,11Z,14Z)/22:6(4Z,7Z,10Z,13Z,16Z,19Z))	C50H80NO8P	854.5664488	9.885166667	Down	Up
pos	P-cresol	C7H8O	109.0649408	3.035333333	Up	Up
pos	Penmacric acid	C7H10N2O5	167.0449934	1.867683333	Down	Down
pos	Wogonin	C16H12O5	285.0752822	4.162116667	Down	Down
neg	(25R)-3beta-hydroxycholest-5-en-7-one-26-oate	C27H42O4	429.2999325	7.57885	Down	Down
neg	{10-Hydroxy-8,8-dimethyl-2-oxo-2H,8H,9H,10H-pyrano[2,3-h] chromen-9-yl} oxidanesulfonic acid	C14H14O8S	363.0172311	3.319883333	Down	Down
neg	{3-Hydroxy-5-oxo-2-[3-(sulfooxy) phenyl]-5H-chromen-7-yl} oxidanium	C15H10O8S	349.0015108	2.530166667	Down	Down
neg	13ʹ-Carboxy-gamma-tocopherol	C28H46O4	445.3313079	8.878566667	Down	Down
neg	13S-hydroxyoctadecadienoic acid	C18H32O3	341.2324911	6.999666667	Down	Down
neg	2,4-Dihydroxybenzoic acid	C7H6O4	153.0178478	2.990866667	Down	Down
neg	2,8-Dihydroxyquinoline-beta-d-glucuronide	C15H15NO8	336.0718474	2.302566667	Down	Down
neg	2-HYDROXY-3,4-DIMETHOXYBENZOIC ACID	C9H10O5	197.0442857	1.80575	Down	Down
neg	2-Phenylethanol glucuronide	C14H18O7	297.0972148	3.811766667	Down	Down
neg	3-(3,5-Dihydroxyphenyl)-1-propanoic acid sulphate	C9H10O7S	261.006705	2.21765	Down	Down
neg	3,3-Dimethylglutaric acid	C7H12O4	159.0648334	3.196816667	Down	Down
neg	3,4,5-Trihydroxy-6-[(6-hydroxy-4-oxo-2-phenyl-4H-chromen-3-yl) oxy] oxane-2-carboxylic acid	C21H18O10	429.0818101	2.7042	Down	Down
neg	3,4,5-Trihydroxy-6-{4-[3-oxo-3-(2,4,6-trihydroxyphenyl) propyl] phenoxy}oxane-2-carboxylic acid	C21H22O11	431.0974625	3.101783333	Down	Down
neg	3b,15b,17a-Trihydroxy-pregnenone	C21H32O4	347.2219707	5.953583333	Up	Up
neg	5-(3ʹ,5ʹ-Dihydroxyphenyl)-gamma-valerolactone 3-O-glucuronide	C17H20O10	365.0843591	3.811766667	Down	Down
neg	5-Nonadecyl-1,3-benzenediol	C25H44O2	421.3316326	8.90155	Down	Down
neg	7a-Hydroxy-5b-cholestan-3-one	C27H46O2	447.34711	9.166916667	Down	Down
neg	7-Ketodeoxycholic acid	C24H38O5	405.2638101	5.741183333	Up	Down
neg	Adrenic acid	C22H36O2	377.2687029	8.20995	Down	Down
neg	Dolicholide	C28H46O6	499.3034023	7.5614	Down	Down
neg	Indole-3-carboxilic acid-O-sulphate	C9H7NO5S	239.9962706	1.80575	Down	Down
neg	LysoPC(O-18:0)	C26H56NO6P	554.3816725	9.07235	Down	up
neg	PC (22:6(4Z,7Z,10Z,13Z,16Z,19Z)/20:4(5Z,8Z,11Z,14Z))	C50H80NO8P	898.5585807	9.865766667	Down	up
neg	Phenyl glucuronide	C12H14O7	269.0660283	2.315033333	Down	up
neg	Polyporusterone B	C28H44O6	497.287582	7.57885	Down	Down
neg	P-Tolyl Sulfate	C7H8O4S	187.0056897	3.015633333	Up	Up
neg	Pyrocatechol sulfate	C6H6O5S	188.9849806	2.062716667	Down	Down

The metabolic pathways significantly enriched by these 47 metabolites were further analyzed using over-representation analysis (ORA) and topological impact calculations. The analysis revealed distinct metabolic pathways in the MCT + Cor group compared to the MCT group, especially in linoleic acid metabolism, alpha-linolenic acid metabolism, ether lipid metabolism, glyoxylate and dicarboxylate metabolism, the citrate cycle (TCA cycle), glycerophospholipid metabolism, glycine, serine, and threonine metabolism, arachidonic acid metabolism, and the biosynthesis of unsaturated fatty acids (Fig. 4E). Among these pathways, linoleic acid metabolism and alpha-linolenic acid metabolism emerged at the most relevant (Fig. 4F).

### Identification of differentially expressed genes and enrichment analysis

**Figure 5:**
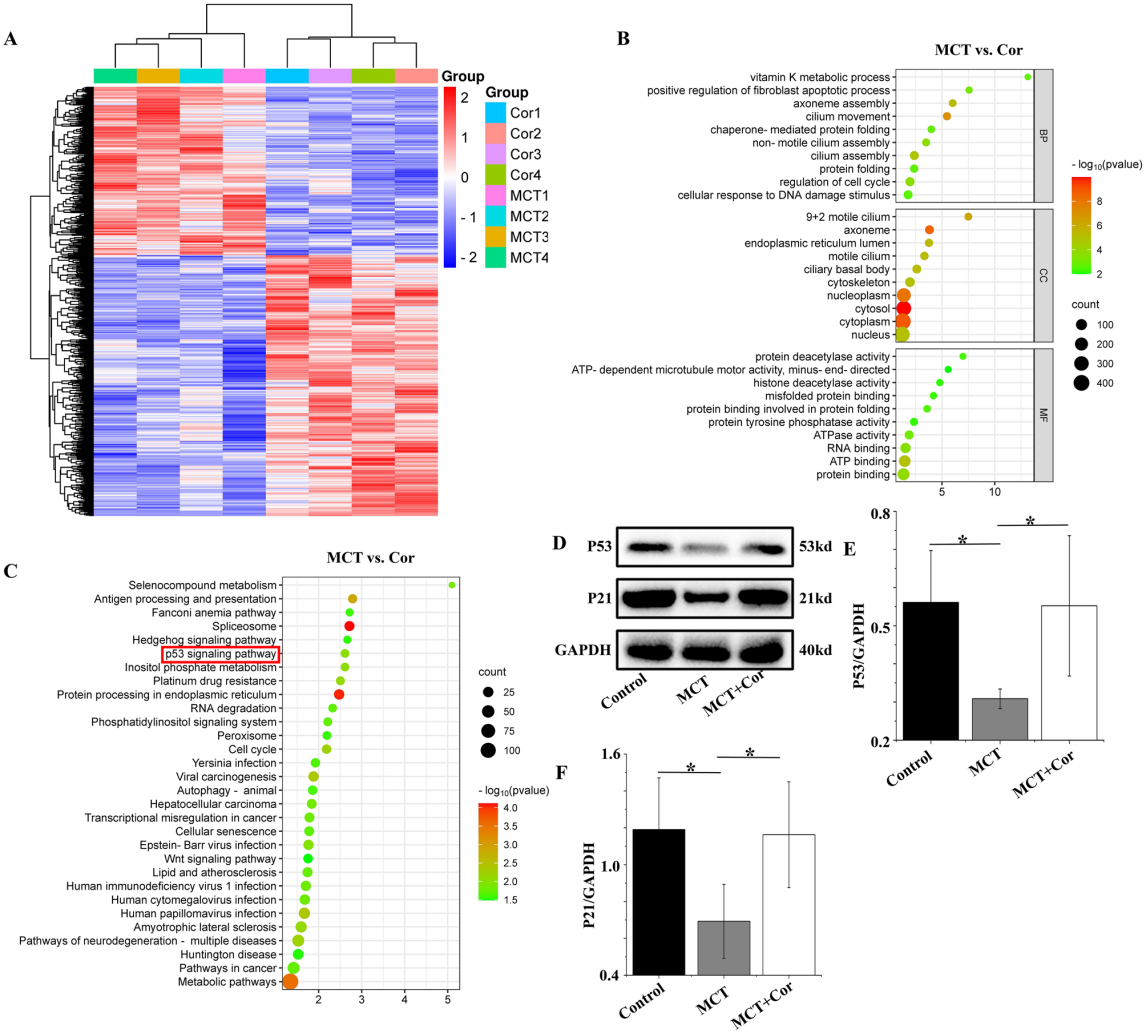
Identification and analysis of differentially expressed genes. (**A**) Differentially expressed mRNA heat map in MCT + Cor and MCT groups; (**B**) GO enrichment bubble map; (**C**) KEGG analysis; (**D**) P53 and P21 protein expression in rat lung tissue; (**E**) Statistical graph of P53 expression in rat lung tissue; (**E**) Statistical graph of P21 expression in rat lung tissue. *p < 0.05.

Analysis of the heat map in Fig. 5A showed that there were 1507 genes differentially expressed between the MCT + Cor and MCT groups (log (FC) > 1, P < 0.05), indicating that Cordycepin improved pulmonary arterial hypertension in rats by altering gene expression. GO analysis revealed that biological processes (BP) were predominantly enriched in activities related to cilia movement, gene filament assembly, cilia assembly, cell cycle regulation, and inactive cilia assembly. Cellular components (CC) were mainly enriched in the cytoplasm, gene filaments, nuclear plasma, and 9 + 2 active cilia. Molecular functions (MF) were associated with ATP binding, protein binding, ribonucleic acid binding, adenosine triphosphatase activity, and protein binding involving protein folding (Fig. 5B). The main pathways identified by KEGG enrichment analysis included selenium compound metabolism, antigen processing and presentation, Fanconi anemia pathway, spliceosome, hedgehog signaling pathway, and the P53 signaling pathway (Fig. 5C). WB results from rat lung tissues showed that MCT exposure reduced the expression of P53 and P21 compared to the Control group. In contrast, Cordycepin treatment led to an increased expression of these proteins in the lungs of rats with PAH (Fig. 5D–F, Supplementary Material 2A). Given the crucial role of the P53 signaling pathway in the pathology of PAH, it is posited that Cordycepin could effectively treat PAH in rats through the activation of the P53–P21 signaling pathway.

### Integrated analysis of transcriptomics and metabolomics

**Figure 6:**
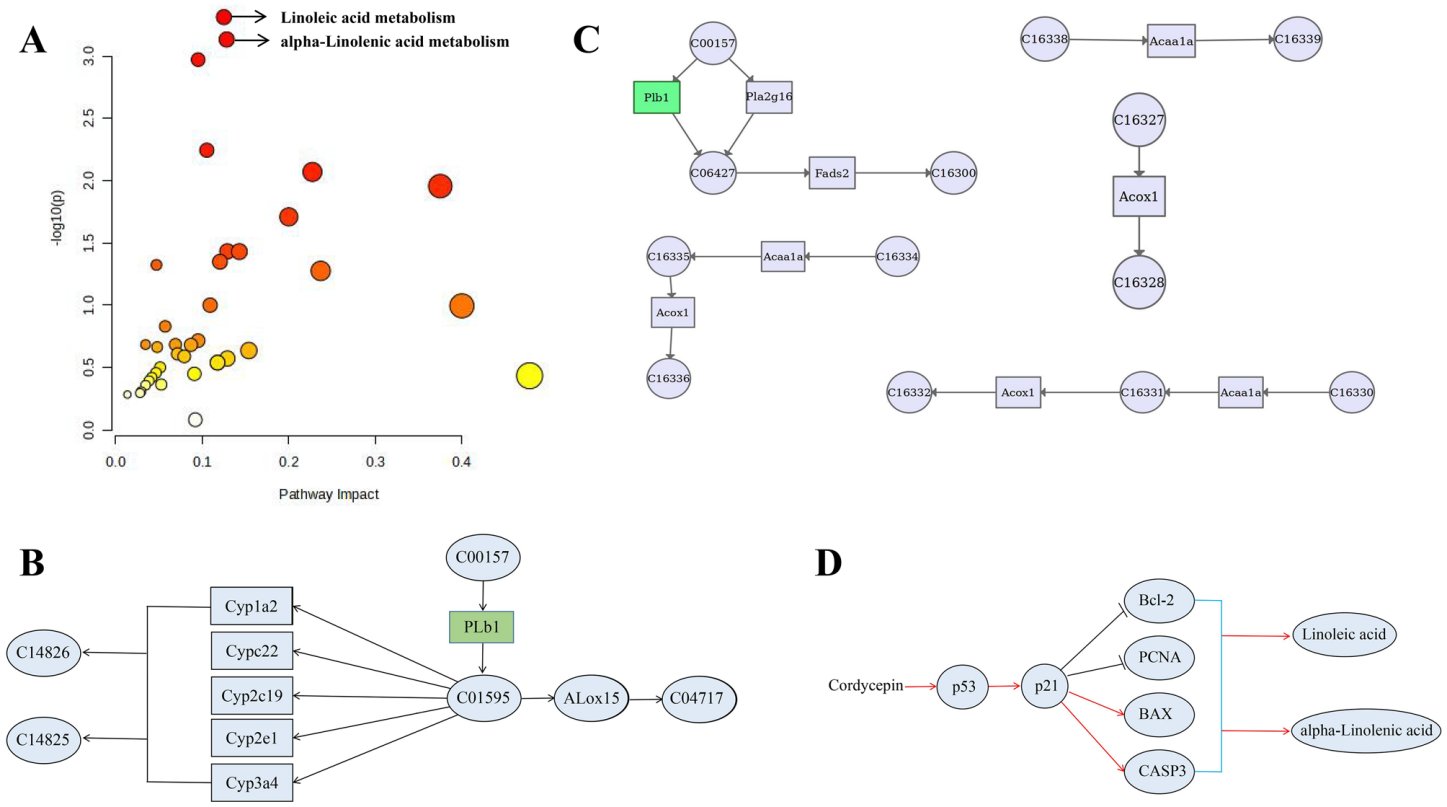
Integrated analysis of transcriptomics and metabolomics. (**A**) Analysis of differentially expressed genes and differentially expressed metabolites associated pathways; (**B**) Schematic diagram of the Linoleic acid metabolic pathway; (**C**) Schematic diagram of the alpha-Linolenic acid metabolism pathway; (**D**) Potential mechanism of Cordycepin in the treatment of PAH.

The MetaboAnalysis tool facilitates the integrated analysis of metabolomics and transcriptomics data, providing a more comprehensive understanding of the effects of Cordycepin in the treatment of pulmonary arterial hypertension (PAH) in rats. The combined data showed that the pathways were primarily enriched in linoleic acid metabolism and alpha-linolenic acid metabolism, in line with previous metabolomics findings. This consistency indicates the critical importance of these metabolic pathways in the treatment of Cordycepin (Fig. 6A). Figure 6B,C showed the specific regulatory mechanisms within the linoleic acid and alpha-linolenic acid metabolism pathways. Our prior network pharmacological experiments identified the P53 pathway as crucial in Cordycepin's action against PAH, and transcriptomics also highlighted its significant role. Thus, we speculated that the linoleic acid metabolism, alpha-linolenic acid metabolism, and the P53 pathway are critical in Cordycepin’s mechanism of action. Literature review further revealed that the P53 protein could regulate both linoleic and alpha-linolenic acid metabolisms. Thus, the hypothesized mechanism by which Cordycepin ameliorates PAH involves the activation of the P53–P21 pathway, which in turn activates CAPS3 and influences the balance between BAX and Bcl-2. This modulation likely increases the expression of linoleic and alpha-linolenic acids, crucial steps in the therapeutic process (Fig. 6D).

### Cordycepin inhibits PD-induced abnormal proliferation and migration of PASMC

**Figure 7:**
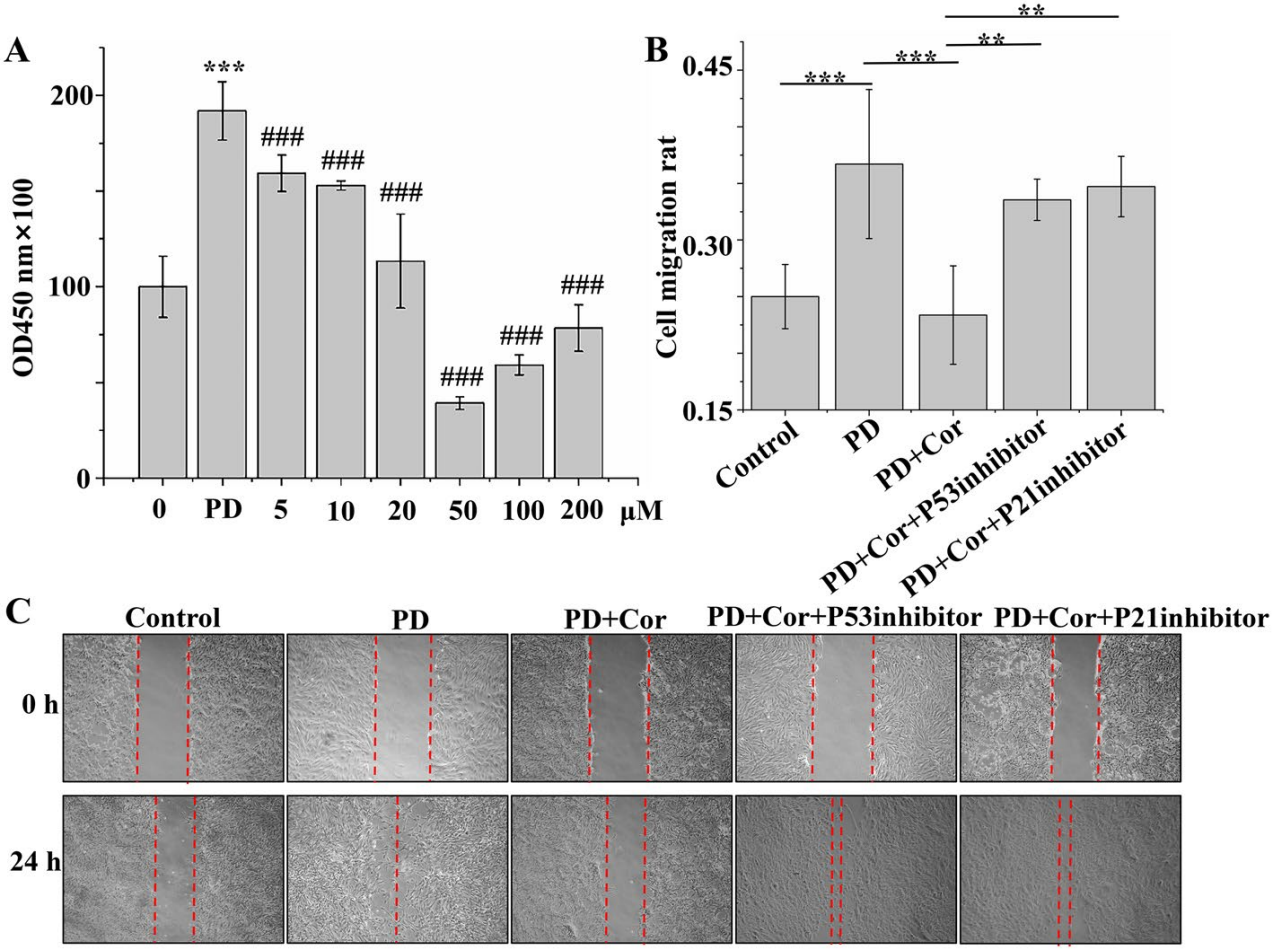
Cordycepin inhibited PD-induced abnormal proliferation and migration of pulmonary artery smooth muscle cells. (**A**) Graph of CCK-8 results (n = 6); * represents PD group vs Control group, # represents PD + Cor group vs PD group; (**B**) Statistical graph of migration area ratio (n = 5). (**C**) Representative images of pulmonary artery smooth muscle cell scratching assay. *PD group vs. Control group. ^#^PD + Cor group vs. MCT group. **p < 0.01, ***p < 0.001. ^###^p < 0.001.

The effects of Cordycepin on PD-induced proliferation of PASMC was assessed using the CCK-8 assay, to further explore Cordycepin's role in pulmonary vascular remodeling in rats with pulmonary arterial hypertension and its underlying mechanisms. PD induction significantly enhanced the proliferation of PASMC compared to the control group, indicating its pro-proliferative effect. Moreover, treatment with Cordycepin significantly inhibited this PD-induced proliferation, with the most significant reduction observed at a concentration of 50 µM (Fig. 7A). Consequently, subsequent studies were conducted using 20 ng/ml PD and 50 µM Cordycepin. Next, the influence of Cordycepin on PASMC migration was investigated using a cell scratching assay. PD exposure significantly increased the migration of PASMCs. However, Cordycepin treatment effectively suppressed this PD-induced migration (Fig. 7B,C). The inhibitory effect of Cordycepin on cell migration could be reversed by inhibitors of P53 and P21, suggesting that the anti-proliferative and anti-migratory effects of Cordycepin are mediated through the P53–P21 signaling pathway.

### Cordycepin inhibits PD-induced abnormal value-added of pulmonary artery smooth muscle cells

**Figure 8:**
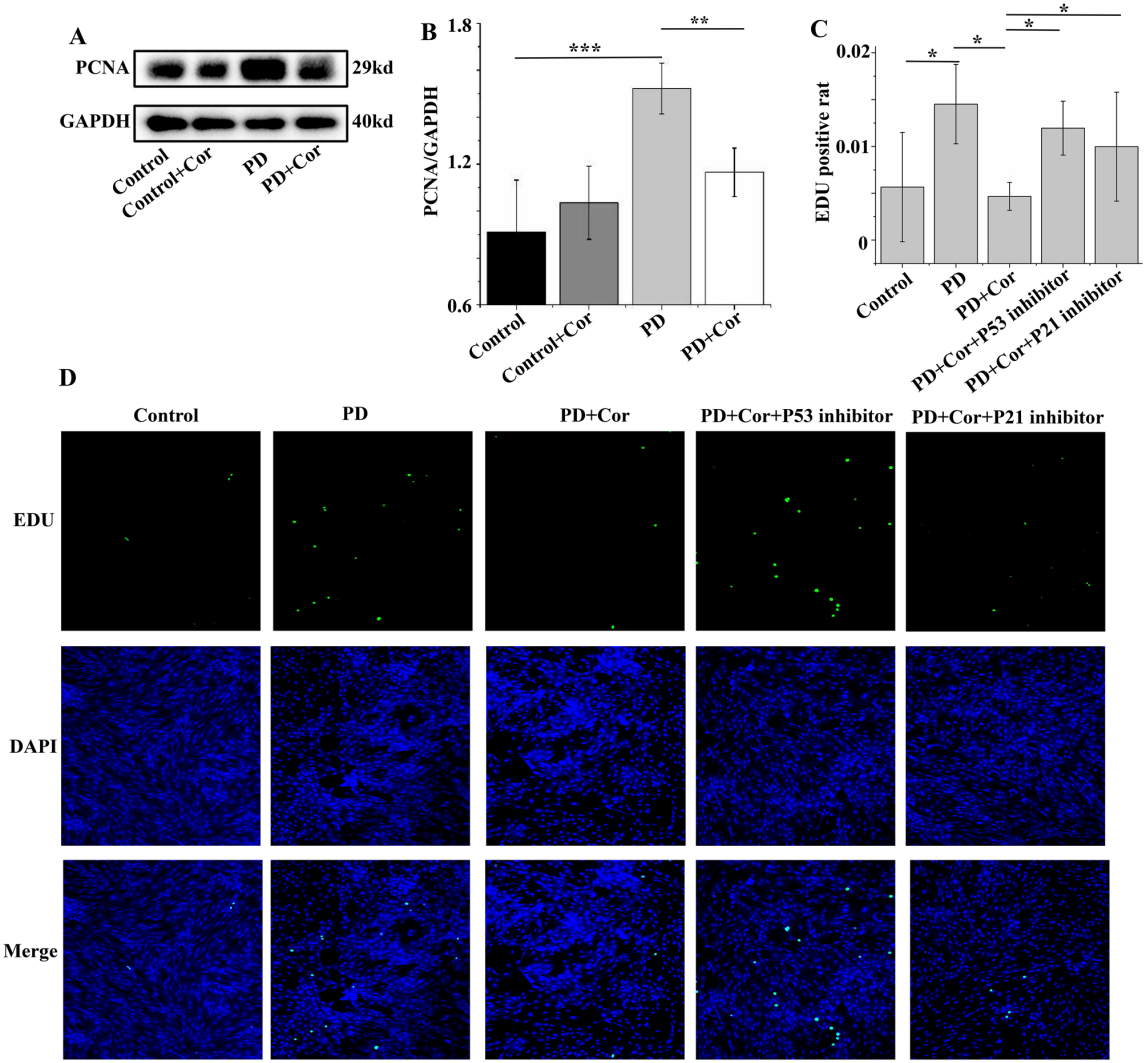
Cordycepin inhibited PD-induced abnormal value-added of pulmonary artery smooth muscle cells. (**A**) Expression of PCNA protein levels; (**B**) Statistical graph of PCNA expression levels; (**C**) Statistical graph of EDU positivity rate (n = 5). (**D**) Representative images of EDU detection in pulmonary artery smooth muscle cells. *p < 0.05, **p < 0.01, ***p < 0.001.

PCNA (Proliferating Cell Nuclear Antigen) is a protein widely utilized as a marker for the proliferation of smooth muscle cells in the pulmonary artery. Compared to the Control group, PD significantly enhanced the expression of PCNA in PASMCs. However, treatment with Cordycepin effectively reduced the PD-induced overexpression of PCNA protein (Fig. 8A,B; Supplementary Material 2B). Additionally, the EDU assay showed that PD significantly promoted DNA synthesis in these cells above levels observed in the Control group. Conversely, Cordycepin significantly inhibited PD-induced DNA synthesis in PASMCs, though this effect was reversed by the introduction of P53 and P21 inhibitors (Fig. 8C,D). These results suggest that Cordycepin's ability to inhibit PD-induced proliferation of pulmonary artery smooth muscle cells is mediated through the P53–P21 signaling pathway.

### Cordycepin promotes apoptosis in pulmonary artery smooth muscle cells

**Figure 9:**
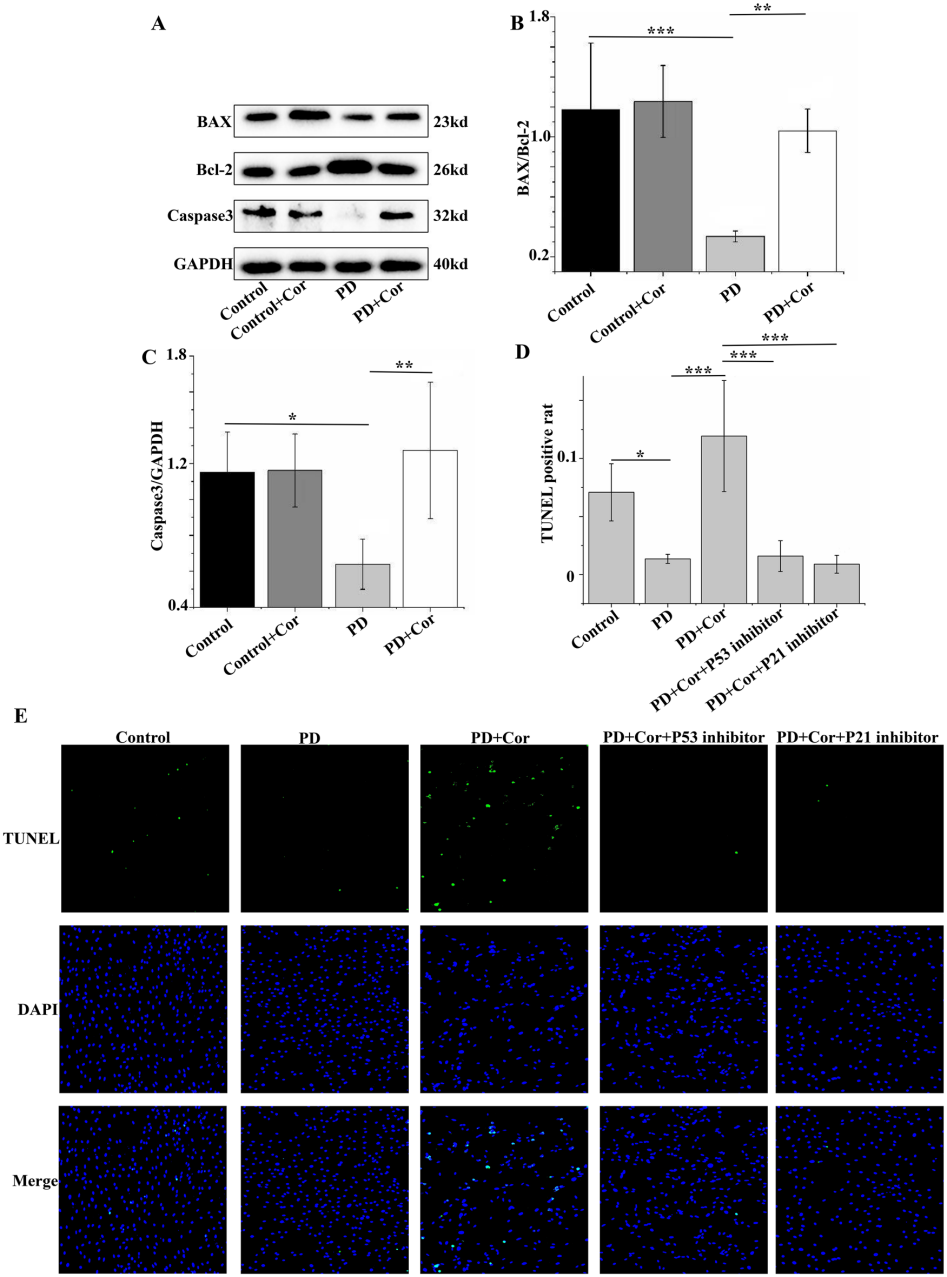
Cordycepin promoted apoptosis in pulmonary artery smooth muscle cells. (**A**) Expression of BAX, Bcl-2, and Caspase3 protein levels. (**B**) Statistical plots of BAX/Bcl-2 expression levels. (**C**) Statistical plots of Caspase3 expression levels. (**D**) Statistical plot of TUNEL positivity rate (n = 5). (**H**) Representative images of TUNEL analysis of pulmonary artery smooth muscle cells. n = 5. *p < 0.05, **p < 0.01, ***p < 0.001.

Proteins such as Caspase 3 and the BAX/Bcl-2 ratio are widely utilized as indicators of apoptosis in PASMCs. In cells stimulated by PD, levels of Caspase 3 and BAX/Bcl-2 were decreased compared to control cells, indicating reduced apoptosis. Conversely, treatment with Cordycepin significantly enhanced the expression of Caspase 3 and BAX/Bcl-2, thereby promoting apoptosis in PASMCs (Fig. 9A–C, Supplementary Material 2B). Additionally, the TUNEL assay further confirmed these findings. TUNEL staining showed that PD exposure diminished apoptosis in PASMCs relative to control cells. However, Cordycepin treatment significantly increased apoptosis in these cells, an effect that was abolished by the addition of P53 and P21 inhibitors (Fig. 9D,E). These findings suggest that Cordycepin enhances apoptosis in PD-stimulated PASMCs primarily through the activation of the P53–P21 signaling pathway.

### Cordycepin activates PD-induced P53–P21 pathway in pulmonary artery smooth muscle cells

**Figure 10:**
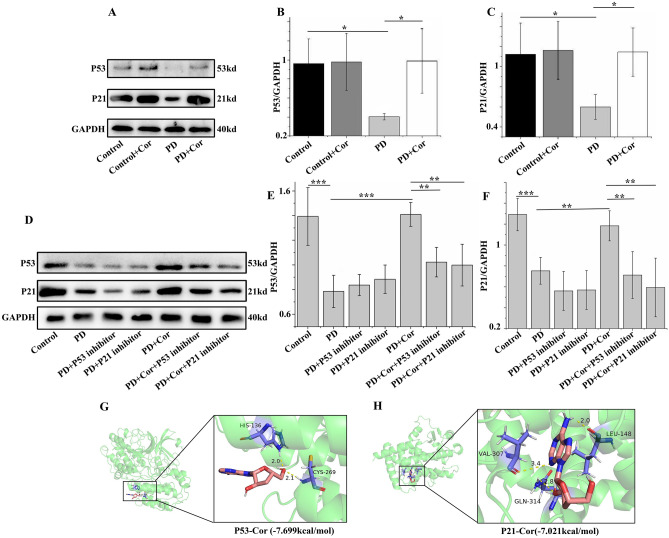
Cordycepin appears to activate the P53–P21 pathway in the PD-induced pulmonary artery smooth muscle cell remodeling. (**A**, **E**) Expression of protein level of P53 and P21; (**B**, **F**) Statistical graph of P53 level; (**C**, **G**) Statistical graph of P21expression level; (**D**) Molecular docking of P53-Cor; (**E**) Molecular docking of P21-Cor. n = 5. *p < 0.05, **p < 0.01, ***p < 0.001.

WB analysis demonstrated that PD exposure leaded to a significant decrease in P53 and P21 protein expression. However, Cordycepin treatment significantly upregulated the expression levels of these critical regulatory proteins (Fig. 10A–C, Supplementary Material 2B). The effectiveness of Cordycepin in enhancing P53 and P21 expression was significantly diminished following the introduction of specific inhibitors for these proteins (Fig. 10D–F, Supplementary Material 2C). Furthermore, molecular docking studies unveiled that the binding energies between Cordycepin and both P53 and P21 proteins, were -7.699 and -7.021 kcal/mol, respectively. these findings collectively suggested a strong molecular interaction between Cordycepin and these proteins (Fig. 10G,H), and Cordycepin’s direct influence on the P53–P21 pathway.

## Discussion

PAH is a chronic, progressive disease affecting the pulmonary vasculature and is characterized by complex pathology. Cordycepin has not previously been reported to have therapeutic effects on PAH. In this study, we utilized an MCT-induced rat model to explore Cordycepin's role in treating PAH. Among the approaches we employed was metabolomic analysis, which helped identify dysregulated metabolites associated with PAH. Furthermore, transcriptomic analyses revealed specific genes and pathways affected by Cordycepin treatment. Cordycepin was found to activate the P53–P21 signaling pathway in PASMCs remodeling induced by PD, suggesting a potential mechanism for its therapeutic action in PAH.

PAH is characterized by pulmonary vasoconstriction, remodeling, inflammation, pulmonary microvascular thrombosis, among other complications^13^. Currently, only limited therapeutic agents are capable of reversing pulmonary vascular remodeling and smooth muscle cell proliferation in PAH. As a result, the development of effective drugs for the treatment of PAH is crucial to reduce mortality and improve the quality of life for PAH patients. Cordycepin, known for its use in treating various chronic and malignant diseases such as leukemia, COPD, and diabetes, has shown a range of beneficial effects including anti-tumor, anti-leukemia, enhancing human immune function, antibacterial, anti-inflammatory, anti-hyperlipidemic, anti-hyperglycemic, and anti-aging effects^14,15^. Cordycepin can be artificially cultured and extracted in large quantities, offering the advantages of potent pharmacological effects and low costs. In this study, MCT was administered intraperitoneally to rats to establish a PAH model. Significant increases in RVSP and RVHI at week 4 post-injection confirmed successful modeling of PAH. Following two weeks of treatment with Cordycepin, both RVSP and RVHI were significantly reduced, indicating that Cordycepin provides therapeutic and preventive benefits for PAH. Furthermore, Cordycepin significantly decreased the thickness of the pulmonary artery walls in rats with PAH, suggesting that it can effectively suppress pulmonary vascular remodeling and reduce pulmonary artery pressure.

Metabolic disorders often play a significant role in the development of PAH. Numerous metabolomic studies have identified significant alterations in the blood metabolite profiles of the patients with PAH^16,17^. In our study, metabolomic results in both positive and negative ion modes demonstrated substantial clustering and metabolic changes post-modeling in the Control, MCT, and MCT + Cor groups, with notable differences in metabolites between the groups. These results suggest that MCT can induce pulmonary metabolic disorders in rats. Compared to the MCT group, the MCT + Cor group showed a trend towards normalization, akin to the control group. Upon analyzing the different metabolites, we found their involvement in lipid metabolism, including oxycholinic acid, taurocholic acid, trihydroxyflavone, and ketodeoxycholic acid. For instance, ileal transposition (IT) surgery has been shown to increase the expression of 3-Oxocholic acid, significantly reducing body weight and fat mass in diabetic GK rats, thereby improving glucose metabolism^18^. Similarly, a high-fat diet supplemented with mutton proteins (HFM) has been found to elevate 12-ketodeoxycholic acid levels, helping to regulate lipid metabolism and ameliorate non-alcoholic fatty liver disease (NAFLD)^19^. These findings are consistent with our experimental results, suggesting that Cordycepin could potentially treat PAH by regulating lipid metabolism through these metabolites. According to KEGG enrichment analysis, Cordycepin primarily alleviated MCT-induced PAH by regulating linoleic acid and alpha-linolenic acid metabolism. Both the linoleic and alpha-linolenic acid metabolisms are implicated in fat accumulation, inflammation, and oxidative stress, all of which may contribute to the pathogenesis of PAH^20,21^.

Transcriptomics, which can reveal differential genes and mechanisms of action, has demonstrated that Cordycepin alleviates PAH in rats by regulating gene expression^22^. Most importantly, KEGG enrichment analysis suggests that the P53 signaling pathway might be central to this modulation. P53 expression is typically reduced in PAH and plays a role in its development^23^. Research by Wei Zhuang et al. highlighted that in MCT-induced PAH in rats, carnitine palmitoyltransferase 1 (CPT 1) was highly expressed, and there was a downregulation of the AMPK-P53–P21 pathway. Furthermore, platelet-derived growth factor (PDGF)-BB increased CPT1 expression in a dose- and time-dependent manner, which promoted proliferation and ATP production in PASMCs while inhibiting the phosphorylation of AMPK, p53, and p21^24^. These results indicate that the P53–P21 pathway is involved in the pathological process of PAH model induced by MCT and PDGF-BB. Therefore, our study utilized the MCT and PDGF-BB induced model to investigate whether Cordycepin ameliorates PAH by regulating the P53–P21 pathway. Research indicates that P21-mediated Bax/Bcl-2/caspase3 signaling can trigger intrinsic apoptosis. The mechanism may involve P21 binding to Bcl-2 family proteins to release Bax through the formation of a P21/Bcl-w complex, promoting cell apoptosis and inhibiting invasion^25,26^. Roninson et al. found that p21 can play dual roles in apoptosis, either inhibiting or promoting cell death by activating apoptotic pathways^27^. These studies suggest a complex relationship between P21 and apoptosis, requiring further exploration. Moreover, some scholars have suggested that P53 may serve as an essential upstream mediator of P21 (WAF1/CIP1) in regulating cellular processes that lead either to growth arrest or to apoptosis^28^. Therefore, we aimed to construct the PAH model to explore whether Cordycepin mediates intracellular apoptosis through the P53–P21 pathway, especially by regulating the expression of Bax/Bcl-2/caspase3, to treat PAH. Western blot results have shown that MCT reduced the expression of P53 and P21 compared to the Control group, whereas Cordycepin treatment increased their expression in rat lung tissues. Based on these observations, Cordycepin is likely to be effective in treating PAH via the P53–P21 signaling pathway.

Integrated analysis of transcriptomics and metabolomics has provided a more comprehensive understanding of the treatment of Cordyceps in PAH rats. Our analysis revealed that metabolic pathways, particularly linoleic acid metabolism and alpha-linolenic acid metabolism, were predominantly enriched. Previous studies have demonstrated that a deficiency in P53 can activate the linoleic acid pathway, leading to a significant increase in linoleic acid expression^29^. Furthermore, it has been observed that in breast cancer cells, the P53–P21WAF1/CIP1, ERK1/2 MAPK, p27KIP1, BRCA1, and NF-κB pathways play critical roles in response to metabolic stress following perturbations in FAS-dependent de novo fatty acid biosynthesis^30^. Our previous network pharmacological experiments have identified the P53–P21 pathway as the most critical apoptotic pathway in Cordycepin’s treatment of PAH. Our molecular docking analysis revealed strong interaction between Cordycepin and both P53 and P21, supporting the significant role of the P53 pathway in the therapeutic mechanisms of Cordycepin against PAH. Based on these findings, we propose that the mechanism by which Cordycepin treats PAH involves the activation of the P53–P21 pathway. This activation further stimulates CAPS3 and influences the balance between BAX and Bcl-2, thereby increasing the expression of linoleic and alpha-linolenic acids which are crucial for managing PAH.

**Figure 11:**
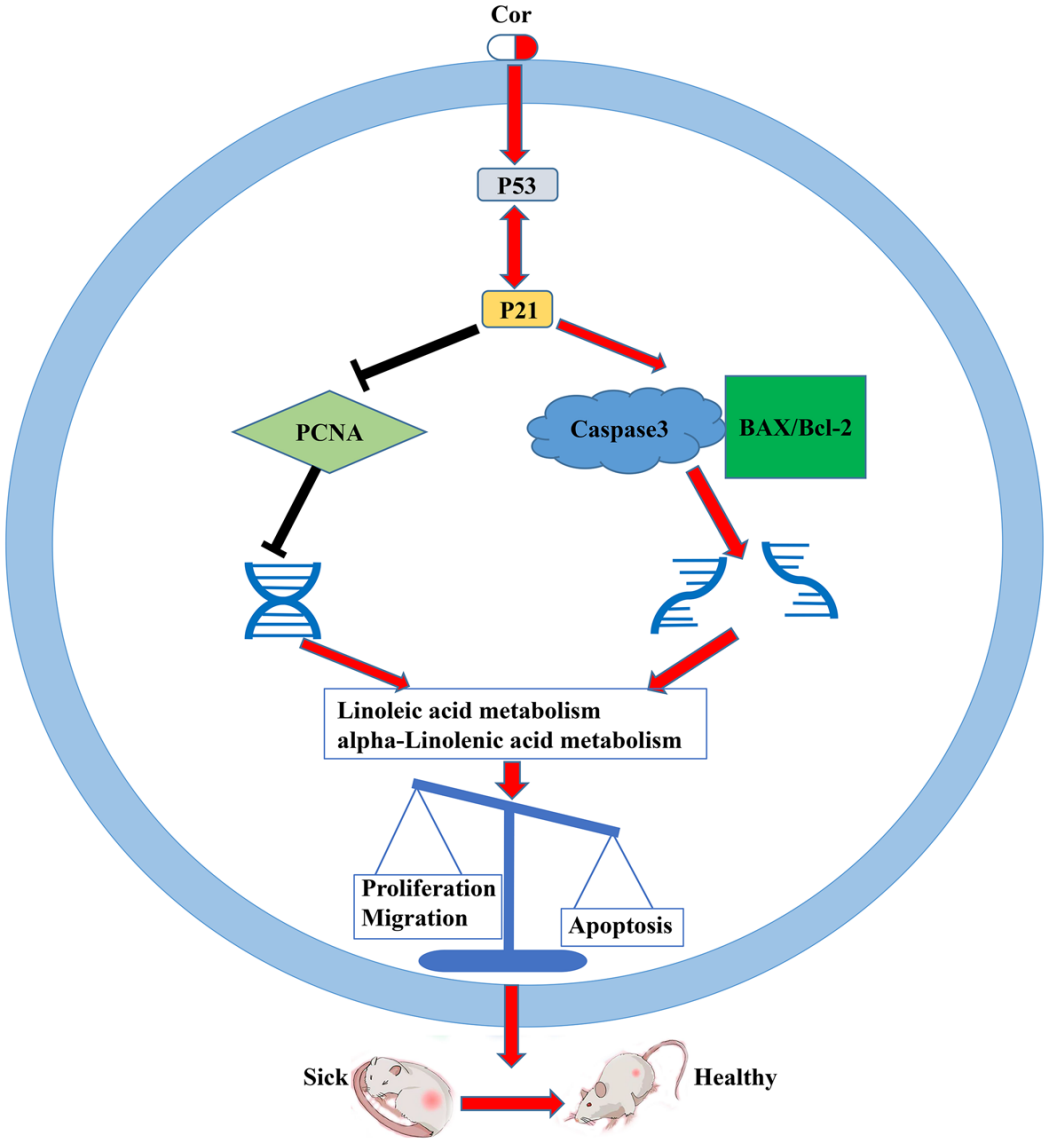
Schematic diagram of the signaling pathway of Cordycepin alleviating PAH and pulmonary vascular remodeling. Cordycepin activates the P53–P21 signal pathway to inhibit the proliferation and migration of PASMCs mediated by PCNA, promote apoptosis mediated by Caspase3, BAX, and Bcl-2, and finally alleviate PAH and pulmonary vascular remodeling.

In the context of PAH, abnormal proliferation and migration of PASMCs are critical factors contributing to the pathogenesis. PCNA, a protein marker commonly used to detect the proliferation of PASMCs, along with the EDU kit, which is used to monitor DNA synthesis in these cells, are vital in studying these processes. Cordycepin has been shown to significantly inhibit PD-induced DNA synthesis in PASMCs; however, this effect was reversed by inhibitors of P53 and P21^31,32^. Apoptosis is considered beneficial in treating PAH as it helps inhibit pulmonary vascular remodeling. An imbalance between pro-apoptotic protein BAX and anti-apoptotic protein Bcl-2 typically leads to reduced apoptosis in PASMCs, exacerbating the disease^33,34^. Caspase3, a key executioner caspase frequently activated during apoptosis^35^, has also been found to be influenced by Cordycepin treatment. In a PD-induced PASMC model, Cordycepin increased Caspase3 expression and the BAX/Bcl-2 ratio, promoting apoptosis. This was supported by TUNEL assay results, indicating that Cordycepin could promote the apoptosis of abnormal pulmonary vascular cells, thereby potentially reducing pulmonary vascular remodeling^36^. In conclusion, Cordycepin may inhibit the abnormal proliferation and migration of PASMCs induced by PD while also promoting apoptosis through the P53–P21 signaling pathway. The activation of this pathway by Cordycepin was confirmed by western blot results, which showed that the activation could be inhibited by P53 and P21 inhibitors. Additionally, molecular docking studies have demonstrated that Cordycepin can effectively bind to P53 and P21 proteins, supporting its potential mechanism of action in treating PAH (Fig. 11)^37^.

## Conclusions

Based on comprehensive data acquired through extensive animal studies, plasma metabolomics, transcriptome sequencing and cytological analysis, this study demonstrates that Cordycepin activates the P53–P21 signaling pathway, which plays a pivotal role in mitigating the progression of PAH. These findings underscore the therapeutic potential of Cordycepin as a promising agent for the treatment of PAH. By modulating key molecular pathways involved in the disease process, Cordycepin offers a viable strategy for effectively reversing the progression of this complex condition.

### Supplementary Information

Supplementary Information 1.

Supplementary Information 2.

## Data Availability

The data used to support the findings of this study are available from https://doi.org/10.4121/20517948.v3.

## References

[1] Hassoun PM (2021). Pulmonary arterial hypertension. N. Engl. J. Med..

[2] Humbert M (2006). Pulmonary arterial hypertension in France: Results from a national registry. Am. J. Respir. Crit. Care Med..

[3] Thenappan T, Ormiston ML, Ryan JJ, Archer SL (2018). Pulmonary arterial hypertension: Pathogenesis and clinical management. BMJ..

[4] Vazquez Z, Klinger JR (2020). Guidelines for the treatment of pulmonary arterial hypertension. Lung..

[5] Das SK, Masuda M, Sakurai A, Sakakibara M (2010). Medicinal uses of the mushroom cordyceps militaris: Current state and prospects. Fitoterapia..

[6] Nakamura K, Shinozuka K, Yoshikawa N (2015). Anticancer and antimetastatic effects of cordycepin, an active component of cordyceps sinensis. J. Pharmacol. Sci..

[7] Yang J, Li YZ, Hylemon PB, Zhang LY, Zhou HP (2017). Cordycepin inhibits LPS-induced inflammatory responses by modulating NOD-like receptor protein 3 inflammasome activation. Biomed. Pharmacother..

[8] Yang SW, Lim L, Ju S, Choi DH, Song H (2015). Effects of matrix metalloproteinase 13 on vascular smooth muscle cells migration via Akt-ERK dependent pathway. Tissue Cell..

[9] Shin S (2009). Role of cordycepin and adenosine on the phenotypic switch of macrophages via induced anti-inflammatory cytokines. Immune Netw..

[10] Zheng Q, Sun J, Li W, Li S, Zhang K (2020). Cordycepin induces apoptosis in human tongue cancer cells in vitro and has antitumor effects in vivo. Arch. Oral Biol..

[11] Cai Z (2018). MiR-125a-5p ameliorates monocrotaline-induced pulmonary arterial hypertension by targeting the TGF-beta1 and IL-6/STAT3 signaling pathways. Exp. Mol. Med..

[12] Su N (2017). Metronomic cordycepin therapy prolongs survival of oral cancer-bearing mice and inhibits epithelial-mesenchymal transition. Molecules..

[13] Galie N (2016). 2015 ESC/ERS Guidelines for the diagnosis and treatment of pulmonary hypertension: The Joint Task Force for the Diagnosis and Treatment of Pulmonary Hypertension of the European Society of Cardiology (ESC) and the European Respiratory Society (ERS): Endorsed by: Association for European Paediatric and Congenital Cardiology (AEPC), International Society for Heart and Lung Transplantation (ISHLT). Eur. Heart J..

[14] Cunningham KG, Manson W, Spring FS, Hutchinson SA (1950). Cordycepin, a metabolic product isolated from cultures of cordyceps Militaris (Linn.) Link. Nature..

[15] Tan, L. *et al*. Anti-inflammatory effects of cordycepin: A review. *Phytother. Res*. (2020). 10.1002/ptr.6890 33090621

[16] Chen C (2020). Metabolomics reveals metabolite changes of patients with pulmonary arterial hypertension in China. J. Cell. Mol. Med..

[17] Swietlik, E. M. *et al*. Plasma metabolomics exhibit response to therapy in chronic thromboembolic pulmonary hypertension. *Eur. Respir. J*. **57**, (2021). 10.1183/13993003.03201-2020 PMC8012591 33060150

[18] Yan K (2019). The changes of serum metabolites in diabetic GK rats after ileal transposition surgery. Obes. Surg..

[19] Ahmad MI (2020). High fat diet incorporated with meat proteins changes biomarkers of lipid metabolism, antioxidant activities, and the serum metabolomic profile in Glrx1(−/−) mice. Food Funct..

[20] Du L (2022). Metabolomic and microbial remodeling by shanmei capsule improves hyperlipidemia in high fat food-induced mice. Front. Cell Infect. Microbiol..

[21] Zhang K (2022). Integrating metabolomics and network pharmacology to reveal the mechanisms of delphinium brunonianum extract against nonalcoholic steatohepatitis. J. Ethnopharmacol..

[22] Jiang YH (2021). Banxia baizhu tianma decoction attenuates obesity-related hypertension. J. Ethnopharmacol..

[23] Hennigs JK (2021). PPARgamma-p53-mediated vasculoregenerative program to reverse pulmonary hypertension. Circ. Res..

[24] Zhuang W (2019). CPT1 regulates the proliferation of pulmonary artery smooth muscle cells through the AMPK-P53–P21 pathway in pulmonary arterial hypertension. Mol. Cell Biochem..

[25] Li J (2022). d-Borneol enhances cisplatin sensitivity via p21/p27-mediated S-phase arrest and cell apoptosis in non-small cell lung cancer cells and a murine xenograft model. Cell Mol. Biol. Lett..

[26] Liu Y (2014). The proapoptotic effect of formononetin in human osteosarcoma cells: Involvement of inactivation of ERK and Akt pathways. Cell Physiol. Biochem..

[27] Roninson IB (2003). Tumor cell senescence in cancer treatment. Cancer Res..

[28] Kagawa S (1997). p53 expression overcomes p21WAF1/CIP1-mediated G1 arrest and induces apoptosis in human cancer cells. Oncogene..

[29] Ju Z (2021). Transcriptomic and metabolomic profiling reveal the P53-dependent benzeneacetic acid attenuation of silica-induced epithelial-mesenchymal transition in human bronchial epithelial cells. Cell Biosci..

[30] Menendez JA, Mehmi I, Atlas E, Colomer R, Lupu R (2004). Novel signaling molecules implicated in tumor-associated fatty acid synthase-dependent breast cancer cell proliferation and survival: Role of exogenous dietary fatty acids, p53–p21WAF1/CIP1, ERK1/2 MAPK, p27KIP1, BRCA1, and NF-kappaB. Int. J. Oncol..

[31] Huang L (2022). Notopterol attenuates monocrotaline-induced pulmonary arterial hypertension in rat. Front. Cardiovasc. Med..

[32] Huang CX (2022). The MFF-SIRT1/3 axis, regulated by miR-340-5p, restores mitochondrial homeostasis of hypoxia-induced pulmonary artery smooth muscle cells. Lab. Invest..

[33] Nisbet RE (2010). Rosiglitazone attenuates chronic hypoxia-induced pulmonary hypertension in a mouse model. Am. J. Respir. Cell Mol. Biol..

[34] Durdu S (2012). Apoptotic vascular smooth muscle cell depletion via BCL2 family of proteins in human ascending aortic aneurysm and dissection. Cardiovasc. Ther..

[35] Deng M (2020). Involvement of P53, P21, and caspase-3 in apoptosis of coronary artery smooth muscle cells in a Kawasaki vasculitis mouse model. Med. Sci. Monit..

[36] Li SS, Liang S, Long Y, Chen X, Jin X (2022). Hsa_circWDR37_016 regulates hypoxia-induced proliferation of pulmonary arterial smooth muscle cells. Cardiovasc. Ther..

[37] Wang S (2022). Utilizing network pharmacology and molecular docking integrated surface plasmon resonance technology to investigate the potential targets and mechanisms of Tripterygium wilfordii against pulmonary artery hypertension. Evid. Based Complement. Alternat. Med..

