# Supplementary material for: Mechanisms of cordycepin in the treatment of pulmonary arterial hypertension in rats based on metabonomics and transcriptomics
Source: Sci Rep. 2024 May 30;14:12431. doi: 10.1038/s41598-024-62163-3 (PMC11139979; doi:10.1038/s41598-024-62163-3)
Supplement: Supplementary file 1 — Supplementary Information 1. [file 41598_2024_62163_MOESM1_ESM.pdf]

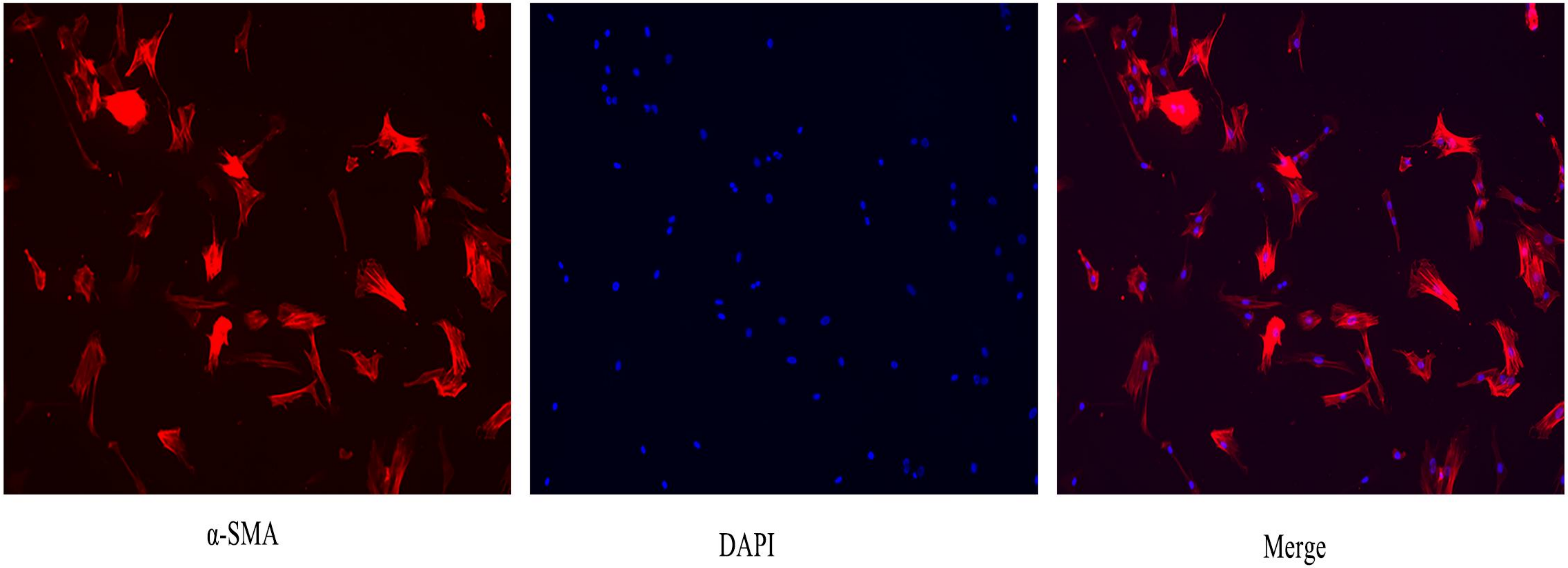

Supplementary Material 1 Identification of PASMCs by fluorescence. Red represents α-SMA. Blue represents PASMC nucleus.
