# Supplementary material for: Mechanisms of cordycepin in the treatment of pulmonary arterial hypertension in rats based on metabonomics and transcriptomics
Source: Sci Rep. 2024 May 30;14:12431. doi: 10.1038/s41598-024-62163-3 (PMC11139979; doi:10.1038/s41598-024-62163-3)
Supplement: Supplementary file 2 — Supplementary Information 2. [file 41598_2024_62163_MOESM2_ESM.pdf]

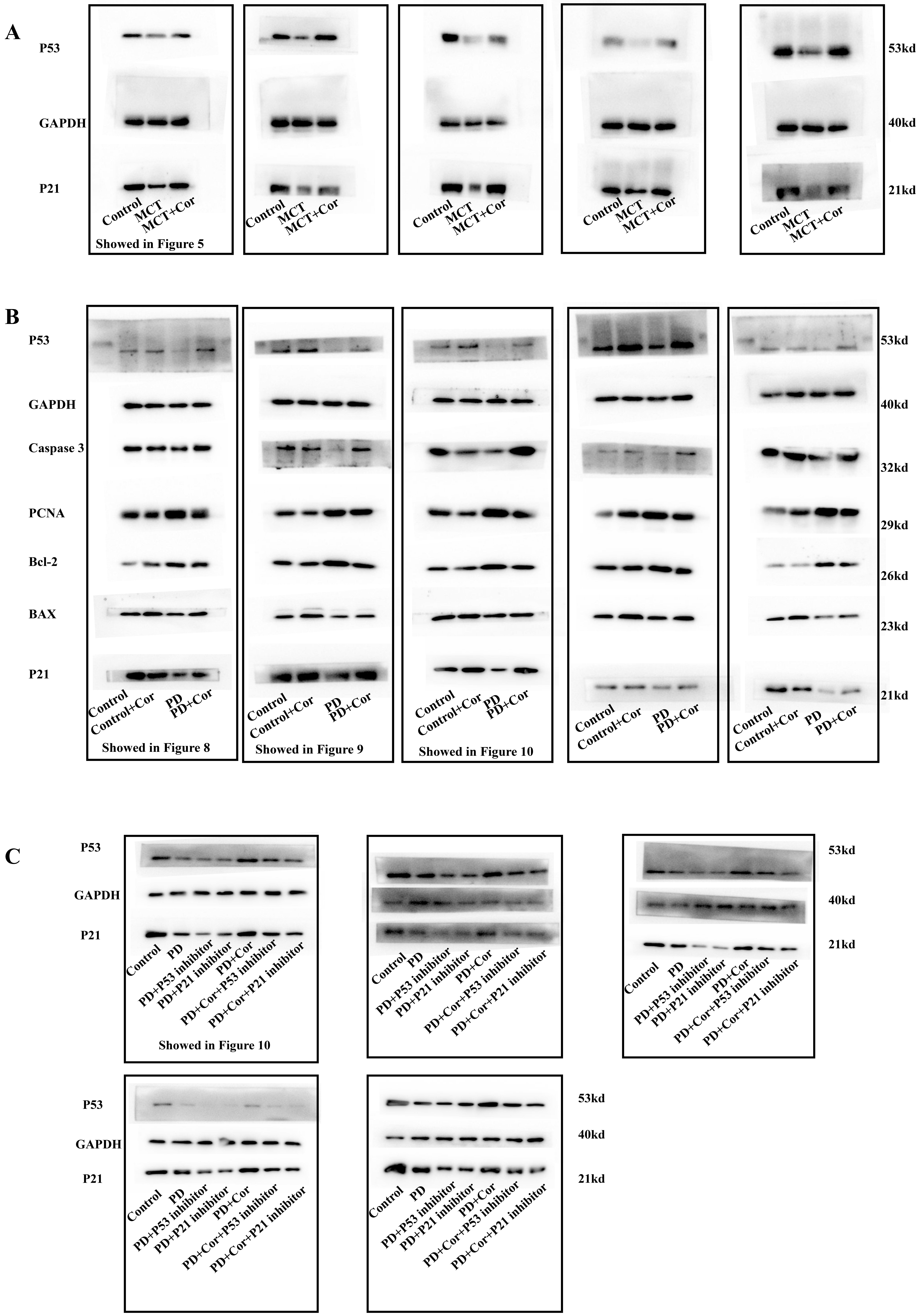

Supplementary Material 2 WB figures of rats and PASMC. (A) WB figures of rats(n=5); (B) WB figures of PASMC (n = 5); (C) WB figures of PASMC with inhibitor(n = 5). Note: The blots were cut prior to hybridisation with antibodies. In order to save time and antibodies, we cut the blots according to the protein size of the antibody instructions, then hybridize the cut strip with the target antibody, discard the blank blot, and then perform secondary antibody incubation and development. So we can't provide a complete blots. We ensure that all experiments are carried out in strict accordance with the correct procedures and repeated 5 times.
